# A feasibility study of a modified treatment strategy combined external beam radiation therapy and brachytherapy for cervical cancer

**DOI:** 10.1002/acm2.13621

**Published:** 2022-04-21

**Authors:** Qi Fu, Wei Li, Jing Zuo, Xi Yang, Yingjie Xu, Manni Huang, Jusheng An, Shuangzheng Jia, Lingying Wu

**Affiliations:** ^1^ Department of Radiation Oncology, National Cancer Center/National Clinical Research Center for Cancer/Cancer Hospital Chinese Academy of Medical Sciences and Peking Union Medical College Beijing China

**Keywords:** brachytherapy, central shielding, cervical cancer, external beam radiation therapy, midline block, volumetric‐modulated arc therapy

## Abstract

**Purpose:**

To evaluate the feasibility of a modified treatment strategy combined external beam radiation therapy (EBRT) and brachytherapy (BT) for cervical cancer through a dosimetry analysis.

**Material and methods:**

This study retrospectively selected 12 cervical cancer patients treated with the conventional treatment strategy, which consisted of 45─50 Gy/25 fractions of EBRT using volumetric‐modulated arc therapy (VMAT) and image‐guided BT with a fraction dose of 5─7 Gy. The modified treatment strategy decreased the central EBRT dose while increasing the number of BT fractions. New target volumes were additionally contoured, and new VMAT EBRT plans were generated for the modified treatment strategy. The dosimetric parameters for evaluation included the doses to the most irradiated 2 cc (D2cc) of the organs at risk (OARs) and doses to at least 90% (D90) of the gross tumor volume (GTV) and high‐risk clinical target volume (HR‐CTV). The total doses to OARs and targets obtained by adding the equivalent doses in 2 Gy fraction (EQD2) from the EBRT and BT plans were used for quantitative comparison between the modified and conventional treatment strategies.

**Results:**

Comparison to the conventional treatment strategy, the modified treatment strategy resulted in a higher bladder D2cc, a slightly lower rectal D2cc and a similar HR‐CTV D90, all with no significant differences (*p* > 0.05). The GTV D90 of the modified treatment strategy was significantly higher than that of the conventional treatment strategy (*p* < 0.01).

**Conclusion:**

The modified treatment strategy can significantly increase the BT dose while remaining the total doses to the bladder and rectum basically unchanged, demonstrating its feasibility and promising prospect in clinical use.

## 1 INTRODUCTION

Radiation therapy is a main treatment for patients with locally advanced cervical cancer, which typically consists of external beam radiation therapy (EBRT) and brachytherapy (BT). The EBRT part of treatment aims to treat the whole pelvis, including the entire cervix, lymph nodes at risk, and a part of bladder and rectum. Recommended EBRT dose is 45–50 Gy in 1.8–2.0 Gy/fraction. The BT part of treatment serves as a boost to EBRT, aiming to irradiate the residual tumor with multiple fractions. According to published guidelines,^1^ the total dose from EBRT and BT should reach at least 80–87 Gy (EQD2, expressed as equivalent dose in 2 Gy fraction) for effective local tumor control. Meanwhile, the minimum cumulative dose from EBRT and BT to the most irradiated 2 cc (D2cc) should not exceed 80–90 Gy (EQD2) and 65–75 Gy (EQD2) for bladder and rectum, respectively. Although BT is used as adjuvant therapy after EBRT, we typically want significant prescription dose (PD) could be delivered with BT because of its ability to provide high localized dose to tumor and extremely steep dose gradients to spare of normal tissues. However, the conventional EBRT treatment to the whole pelvis already results in high doses to bladder and rectum (even higher than PD due to nonuniform dose distributions). In order to reduce radiological toxicity, the BT dose usually cannot be increased any more.

Aiming at this problem, many treatment centers used to place a midline block in external irradiation fields to reduce the EBRT dose in the midline region so that the significant dose could be delivered with BT.^2^, ^3^, ^4^ However, this approach only applied to the EBRT using conformal radiation therapy (CRT). Nowadays, intensity‐modulated radiation therapy (IMRT) and volumetric‐modulated arc therapy (VMAT) have displaced CRT as common techniques for EBRT because of superior dose distributions and lower radiological toxicity.^5^, ^6^, ^7^, ^8^, ^9^ To take the advantages of these advanced techniques, we developed a modified treatment strategy using VMAT to decrease the doses to bladder and rectum from EBRT while increasing the dose from BT by adding BT fractions. This study presented this modified treatment strategy, evaluated its dosimetric parameters, and compared it with the conventional treatment strategy.

## 2 METHODS AND MATERIALS

### 2.1 Patient selection and contouring

**FIGURE 1:**
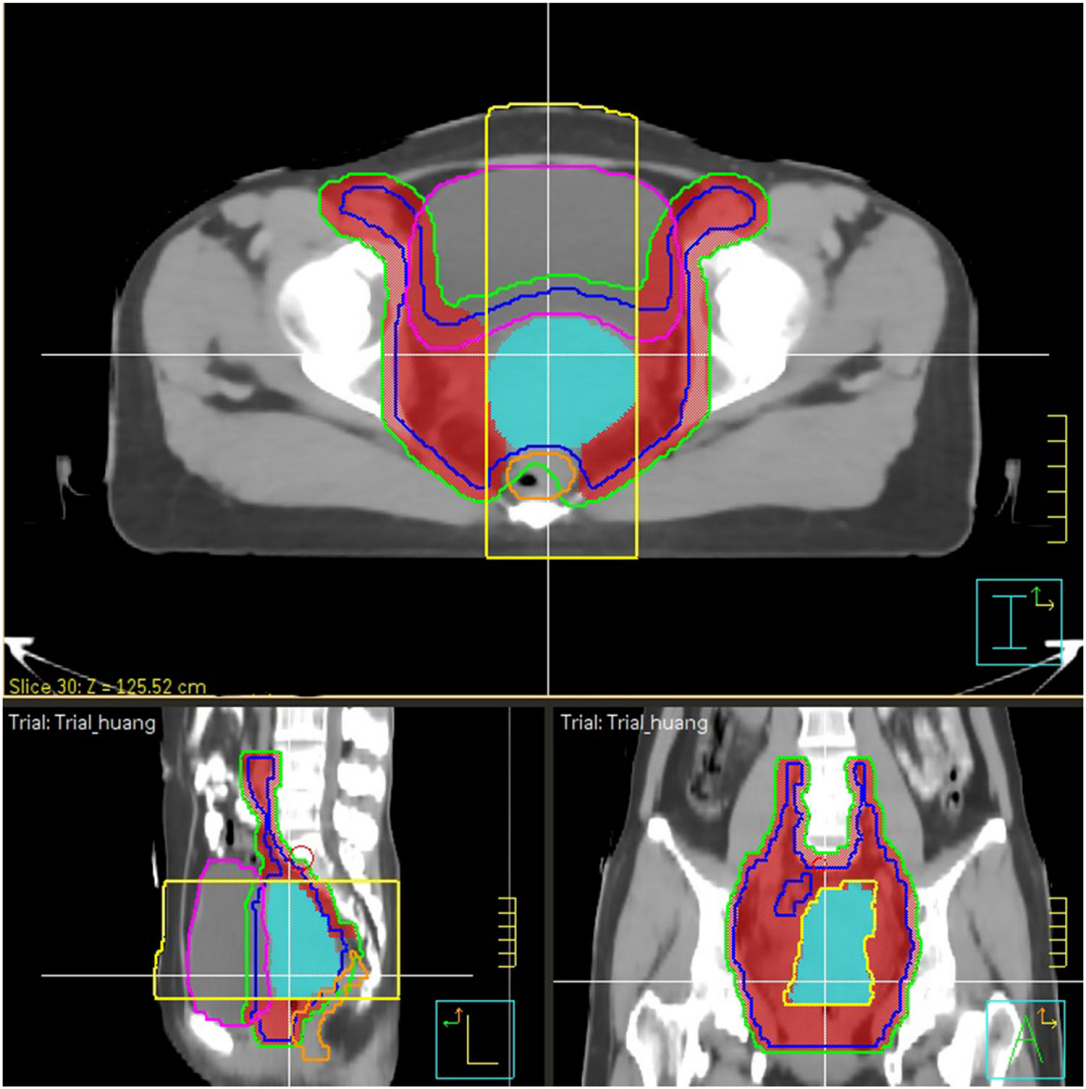
Axial, sagittal, and coronal images showing the clinical target volume (CTV) (blue line), planning target volume (PTV) (green line), PTV_U+C_ (cyan area), PTV‐PTV_U+C_ (red area), bladder (pink line), rectum (orange line), and the scope of D2cc evaluation (yellow line)

This study included 12 cervical cancer patients treated with VMAT EBRT and more than three fractions of CT‐guided high‐dose‐rate (HDR) BT using tandem/ovoid (T/O) applicators or T/O applicators with interstitial needles. According to the International Federation of Gynecology and Obstetrics stage classification, the local tumor stages of the patients were from stage Ib2 to IIIC2r. All patients underwent CT scans using a Brilliance CT Big Bore (Philips, Amsterdam, Netherlands) with 5‐mm slice thickness for EBRT and 3‐mm slice thickness for BT, respectively. The clinical target volume (CTV) of EBRT consists of the gross tumor volume (GTV), whole uterus and cervix, at least the upper half of the vagina, bilateral parametrium, uterosacral ligaments, and lymph nodal region at risk. A 5‐mm expansion was applied to the CTV to create the planning target volume (PTV). Besides, our modified EBRT needs to additionally contour a PTV_U+C_ and a PTV‐PTV_U+C_. The PTV_U+C_ consists of the uterus, cervix, and the top of the vagina, aiming to include most regions the BT dose could cover. The PTV‐PTV_U+C_ is the remaining part of the PTV after subtracting the PTV_U+C_ and adjacent bladder and rectum, as shown in Figure 1. The target volumes of BT include GTV and high‐risk CTV (HR‐CTV), both contoured in accordance with the Groupe Européen de Curiethérapie–European Society for Therapeutic Radiology and Oncology recommendation.^10^ Normal tissues including the bladder, rectum, sigmoid, colon, intestine, right and left femoral heads, and pelvic bones were also contoured for planning and dose evaluation.

### 2.2 Prescriptions

The PDs of the conventional EBRT were 45–50 Gy to the PTV and a boost dose of 10–15 Gy to the enlarged lymph nodes in 25 fractions. To spare the bladder and rectum, we reduced the number of fractions from 25 to 21 and decreased the irradiation dose to the PTV_U+C_. The PDs of the modified EBRT were 35.7 Gy to the PTV_U+C_ and 45.15 Gy to the PTV‐PTV_U+C_. Enlarged lymph nodes still received extra boost dose of 10–15 Gy. For the BT part of treatment, the PDs to HR‐CTV were 5–7 Gy/fraction. The difference between the modified BT and the conventional BT is the number of fractions. We increase the number of the modified BT fractions from 4 to 6. All the PDs are listed in Table 1. Despite the change in the EBRT and BT fraction numbers, the modified strategy can be implemented in much the same way as the conventional strategy. The EBRT is still given in five fractions a week. The HDR BT is performed weekly for 6 consecutive weeks. Often the BT is initiated during the last week of EBRT, when primary tumor regression has been sufficient to permit BT application insertion. Before BT initiation, MRI scanning will be performed to confirm this indication and to aid for target contouring. To lighten the workload of BT treatment, CT‐guided BT is performed every two fractions in our institution. In the latter of the two fractions, we repeat the former application insertion and deliver the former BT plan again without CT guide. That is to say, each BT plan will be delivered twice, no matter in the modified or conventional treatment procedures.

**TABLE 1:** The prescription doses and the corresponding equivalent doses in 2 Gy fraction (EQD2) of the modified and conventional treatment strategies

Treatment	Target	PD per fraction (Gy)	Fractions	PD (Gy)	EQD2 (Gy)
Modified EBRT	PTV_U+C_	1.7	21	35.7	34.81
	PTV‐PTV_U+C_	2.15	21	45.15	45.71
	Lymph nodes	2.6–2.8	21	54.6–58.8	57.33–62.72
Modified BT	HR‐CTV	5–7	6	30–42	37.5–59.5
Conventional EBRT	PTV	1.8–2	25	45–50	44.25–50
	Lymph nodes	2.2–2.4	25	55–60	55.92–62
Conventional BT	HR‐CTV	5–7	4	20–28	25–39.67

Abbreviations: BT, brachytherapy; EBRT, external beam radiation therapy; HR‐CTV, high‐risk clinical target volume; PD, prescription doses; PTV, planning target volume.

### 2.3 Treatment planning

All EBRT VMAT plans were designed using Pinnacle v9.1─16.2 (Philips Radiation Oncology Systems, Fitchburg, WI, USA) and delivered using double full arcs with 6‐MV X‐ray. Dose constraints for normal tissues were as follows: rectum: Dmax < 52 Gy, volume receiving > 50 Gy (V50) < 20%; sigmoid: Dmax < 52 Gy, V40 < 60%; bladder: V50 < 20%; intestines: Dmax < 52 Gy, V40 < 50%; spinal cord: Dmax < 40 Gy; femoral heads: V30 < 30%, pelvic bone marrow: V30 < 50%; kidneys: V20 < 20%. For the modified EBRT plans, the bladder and rectum were limited with stricter doses because of the decrease in the central dose. All plans were normalized to ensure that 95% of the target volumes received 100% of the PD.

The BT planning was performed using Oncentra Brachy v4.6 (Elekta Brachytherapy, Veneedal, The Netherlands), with a ^192^Ir source for a Flexitron afterloader unit. The activation step was set to 2 mm. The source dwell times for each BT plan were optimized by hybrid inverse planning optimization algorithm and manually adjusted. The dose constraints used for optimization followed the National Comprehensive Cancer Network clinical practice guidelines.^5^ All the BT plans were normalized so that 90% of the HR‐CTV (D90) received 100% of the prescribed fraction dose. Treatment planning in this study was performed by a single experienced planner.

### 2.4 Dosimetric evaluation

The dosimetric parameters including D2cc of bladder and rectum and D90 of the GTV and HR‐CTV were individually recorded for all plans. The boost doses to the parametrial lymph nodes will not contribute to the BT boost region but may increase the D2cc values. Thus, for both modified and conventional EBRT plans, we only computed the D2cc of central bladder and rectum, which were the overlaps between these organs and the PTV_U+C_ expanded by 5 cm in anterior‐posterior direction, as shown in Figure 1. As the GTV and HR‐CTV were fully contained in the PTV (or PTV_U+C_), for simplicity, their D90 were considered to be the same as the PD of the PTV (or PTV_U+C_). As mentioned above, suppose one BT plan could be implemented two times, then the dose volume parameters of each plan would be counted twice. The modified treatment needed to use three BT plans, and the conventional treatment used first two BT plans, according to their corresponding numbers of fractions. The total doses for evaluation were the sum of doses from both EBRT and BT and normalized to EQD2 using the following formula:

$$\mathrm{EQD}2\;=\;nd\cdot \frac{d+\alpha /\beta }{2+\alpha /\beta }\;,$$

where *n* was the number of fractions, *d* was the fraction dose, and *α* and *β* were the radiosensitivity parameters for the linear‐quadratic responses, respectively. In this study, α/βfor the targets and organs were 10 Gy and 3 Gy, respectively. The statistical significance of the dosimetric results was proven with a two‐sided paired *t*‐test at a 5% level significance level.

## 3 RESULTS

**FIGURE 2:**
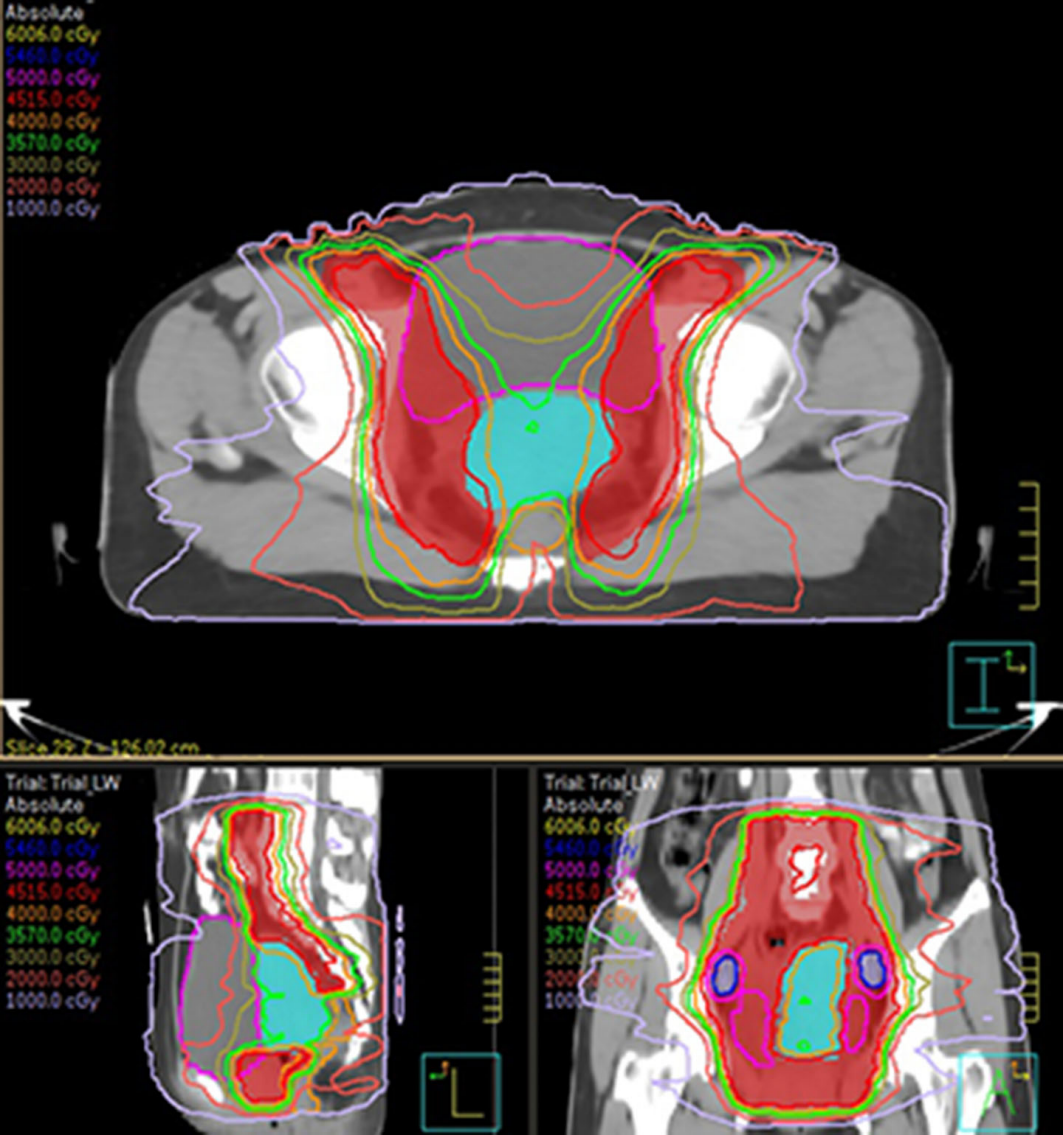
An example of dose distributions for the modified volumetric‐modulated arc therapy (VMAT) external beam radiation therapy (EBRT) plans

Table 2 summarizes the total D2cc of the bladder and rectum and the total D90 of the GTV and HR‐CTV for the modified and conventional treatment strategies. The modified treatment strategy resulted in a slightly higher bladder D2cc (78.33 ± 6.43 Gy vs. 76.80 ± 5.39 Gy), a slightly lower rectal D2cc (64.29 ± 8.26 Gy vs. 65.46 ± 8.38 Gy), and a similar HR‐CTV D90 (80.25 ± 5.67 Gy vs. 79.77 ± 4.18 Gy) in comparison to the conventional treatment strategy. However, these values showed no significant differences (*p* = 0.167, 0.405, and 0.595). Only the GTV D90 of the modified treatment strategy was significantly higher than that of the conventional treatment strategy (*p* < 0.01), which benefitted from the increased number of BT fractions. Figure 2 shows an example of typical dose distribution for the modified EBRT plans.

**TABLE 2:** Comparison of total dosimetric parameters for the 12 patients between the modified and conventional treatment strategies (Gy)

	Bladder D2cc		Rectum D2cc		GTV D90		HR‐CTV D90	
Patient	Modified	Conventional	Modified	Conventional	Modified	Conventional	Modified	Conventional
1	77.05	80.38	66.25	74.84	81.00	83.43	64.52	70.38
2	77.66	74.86	71.53	73.63	107.16	98.70	82.81	82.00
3	83.91	84.39	49.33	53.08	109.18	96.80	88.43	85.75
4	66.49	70.66	69.19	72.51	109.63	99.99	86.64	85.83
5	88.21	82.24	78.42	75.90	86.41	85.86	69.16	71.85
6	83.60	79.86	70.09	70.52	104.46	95.14	82.84	82.04
7	83.28	75.99	67.93	66.23	94.12	89.48	78.16	79.15
8	80.27	81.87	55.29	51.70	94.39	89.63	77.52	78.50
9	84.34	81.59	59.03	66.16	89.53	82.13	75.81	75.00
10	72.83	69.04	52.33	54.05	126.12	112.10	86.64	85.83
11	71.21	68.82	63.80	66.72	125.82	99.75	83.14	76.58
12	71.12	71.86	68.34	60.15	101.31	92.99	79.31	76.25
Mean	78.33 ± 6.43	76.80 ± 5.39	64.29 ± 8.26	65.46 ± 8.38	102.43 ± 13.69	93.83 ± 8.10	80.25 ± 5.67	79.77 ± 4.18
*p* ‐Value	0.167		0.405		0.002		0.595	

Abbreviations: GTV, gross tumor volume; HR‐CTV, high‐risk clinical target volume.

## 4 DISCUSSION

Radiation therapy for cervical cancer is delivered by a combination of EBRT and BT. To fully take its advantage of high local tumor control and significant sparing of normal tissues, the BT dose should be increased as much as possible. Meanwhile, the doses to bladder and rectum from EBRT should be limited to reduce radiological toxicity. In the past, the common approach of achieving this is to use a midline block during EBRT to shield the central bladder and rectum. However, this approach has been in use for decades and unable to adapt to advanced techniques used in EBRT, such as IMRT and VMAT. Currently, there are only two studies attempted to apply these advanced techniques to continue this approach. Macdonald et al. developed a pseudo‐split‐field technique using IMRT based on the concept of midline block.^11^ This technique was proved to be able to limit the central EBRT dose and maximize the BT dose. However, it applied only to IMRT but not VMAT. Later, Tamaki et al. applied a “two‐step” VMAT in EBRT for early‐age cervical cancer, which was likely to reduce radiation doses to rectum and small bowel.^12^ They followed the central‐shielding principle and divided EBRT into two parts: whole pelvic irradiation of 20 Gy/10 fractions and central‐shielding pelvis irradiation of 30 Gy/15 fractions. It meant that two VMAT plans needed to be created for the EBRT due to the change in the targets. In fact, VMAT technique has the ability to generate superior dose distributions with many dose gradients and low dose in center. Therefore, in the modified treatment strategy, we integrated the “two‐step” VMAT to a simpler “one‐step” VMAT with only 21 fractions. From Figure 2, one can see that our required EBRT dose distribution was well achieved using VMAT. Besides, the region shielded by the midline block or pseudo‐split‐field IMRT is in regular shape and located in center. However, it is not always suitable for patients with off‐center uterus or peculiarly shaped tumor. Our modified VMAT EBRT allows radiation oncologists to flexibly adjust the low‐dose target volume (PTV_U+C_) based on the specific shapes of uterus and tumor, or actual treatment needs so as to better spare of the bladder and rectum.

For the conventional treatment strategy, the EBRT dose to pelvis is 45–50 Gy, resulting in the D2cc of bladder and rectum also in the range of 45–50 Gy or sometimes higher than 50 Gy, due to nonuniform dose distributions. Published guidelines recommended that the D2cc of bladder and rectum should be respectively limited to 5.1 Gy and 4.3 Gy per BT fraction and to 80–90 Gy (EQD2) and 65–75 Gy (EQD2) in total. This means that the conventional BT can only have no more than four fractions, which limits the increase of BT dose. In order to increase the number of BT fractions, our modified treatment strategy decreased the EBRT dose to PTV_U+C_ from 45 Gy to 35.7 Gy. But the fraction dose to the PTV_U+C_ for the modified EBRT was only 0.1 Gy lower than that for the conventional EBRT. This kept the radiobiological effect of the modified EBRT on this region as close as possible with that of the conventional EBRT. For the parametrial targets (PTV‐PTV_U+C_) and the involved lymph nodes, we increased their fraction doses so that their EQD2 were similar between the two EBRT. In addition, positioning errors and changes in bladder and rectum filling between EBRT fractions may change the location of the uterus, leading to the bowels and sigmoid colon moving into the target range and receiving high radiation dose. The decrease in the central dose of EBRT may help to lower the incidence of radiation enteritis and colitis caused by these displacements.

Considering image‐guided BT is a labor‐intensive treatment, we suggest to perform image‐guided BT every two fractions and deliver the same BT plan with the same application insertion for these two fractions. We have verified this approach in a few dozen clinical cases that the dose difference between the two fractions could be limited in 5%. With this approach, the conventional strategy needs two fractions of image‐guided BT, and the modified strategy needs three fractions. The resulting increase in the workload for the modified BT treatment is acceptable. Thanks to the increased number of BT fractions, our strategy significantly increased the total D90 of the GTV while remaining the total D2cc of the bladder and rectum basically unchanged. This indicates that the modified strategy is likely to improve the local tumor control without an increase in the risk of radiological toxicity. However, this is just a dosimetric study of this modified treatment strategy. The clinical usability and functional outcome of this strategy should be further investigated in future.

## 5 CONCLUSION

This study showed the dosimetric advantages of our modified treatment strategy for cervical cancer, demonstrating that it was feasible in clinical use. We therefore encourage other groups to test this strategy in clinical trials with us in future.

## CONFLICT OF INTEREST

The authors declare that there is no conflict of interest that could be perceived as prejudicing the impartiality of the research reported.

## AUTHOR CONTRIBUTIONS

*Conception and design of the study* : Qi Fu, Wei Li, and Jing Zuo. *Delineation and assessment of treatment*: Wei Li, Xi Yang, Manni Huang, Jusheng An, and Shuangzheng Jia. *Acquisition and collection of data*: Qi Fu and Wei Li. *Analysis and interpretation of data*: Qi Fu, Yingjie Xu, Jing Zuo, and Xi Yang. *Writing and revising the paper*: Qi Fu, Yingjie Xu, Jing Zuo, and Xi Yang. *Final approval of the manuscript*: Yingjie Xu, Jing Zuo, and Lingying Wu.

## References

[1] Koh WJ , Abu‐Rustum NR , Bean S , et al. Cervical cancer, version 3.2019, NCCN clinical practice guidelines in oncology. J Natl Compr Canc Netw. 2019;17:64‐84. 10.6004/jnccn.2019.0001 30659131

[2] Wolfson AH , Abdel‐Wahab M , Markoe AM , et al. A quantitative assessment of standard vs. customized midline shield construction for invasive cervical carcinoma. Int J Radiat Oncol Biol Phys. 1997;37:237‐242. 10.1016/s0360-3016(96)00469-5 9054901

[3] Potter R , Knocke TH , Fellner C , et al. Definitive radiotherapy based on HDR brachytherapy with iridium 192 in uterine cervix carcinoma: report on the Vienna University Hospital findings (1993–1997) compared to the preceding period in the context of ICRU 38 recommendations. Cancer Radiother. 2000;4:159‐172. 10.1016/S1278-3218(00)88900-3 10812362

[4] Huang EY , Lin H , Hsu HC , et al. High external parametrial dose can increase the probability of radiation proctitis in patients with uterine cervix cancer. Gynecol Oncol. 2000;79(3):406‐410. 10.1006/gyno.2000.5997 11104610

[5] Mundt AJ , Lujan AE , Rotmensch J , et al. Intensity‐modulated whole pelvic radiotherapy in women with gynecologic malignancies. Int J Radiat Oncol Biol Phys. 2002;52:1330‐1337. 10.1016/s0360-3016(01)02785-7 11955746

[6] Hasselle MD , Rose BS , Kochanski JD , et al. Clinical outcomes of intensity‐modulated pelvic radiation therapy for carcinoma of the cervix. Int J Radiat Oncol Biol Phys. 2011;80:1436‐1445. 10.1016/j.ijrobp.2010.04.041 20708346

[7] Chen MF , Tseng CJ , Tseng CC , et al. Clinical outcome in posthysterectomy cervical cancer patients treated with concurrent Cisplatin and intensity‐modulated pelvic radiotherapy: comparison with conventional radiotherapy. Int J Radiat Oncol Biol Phys. 2007;67:1438‐1444. 10.1016/j.ijrobp.2006.11.005 17394944

[8] Sharfo AW , Voet PW , Breedveld S , et al. Comparison of VMAT and IMRT strategies for cervical cancer patients using automated planning. Radiother Oncol. 2015;114:395‐401. 10.1016/j.radonc.2015.02.006 25725503

[9] Marnitz S , Wlodarczyk W , Neumann O , et al. Which technique for radiation is most beneficial for patients with locally advanced cervical cancer? Intensity modulated proton therapy versus intensity modulated photon treatment, helical tomotherapy and volumetric arc therapy for primary radiation—an intraindividual comparison. Radiat Oncol. 2015;10:91. 10.1186/s13014-015-0402-z 25896675 PMC4404108

[10] Pötter R , Haie‐Meder C , Limbergen EV , et al. Recommendations from gynaecological (GYN) GEC ESTRO working group (II): concepts and terms in 3D image‐based treatment planning in cervix cancer brachytherapy‐3D dose volume parameters and aspects of 3D image‐based anatomy, radiation physics, radiobiology. Radiother Oncol. 2006;78(1):67‐77. 10.1016/j.radonc.2004.12.015 16403584

[11] Macdonald DM , Lin LL , Biehl K , et al. Combined intensity‐modulated radiation therapy and brachytherapy in the treatment of cervical cancer. Int J Radiat Oncol Biol Phys. 2008;71(2):618‐624. 10.1016/j.ijrobp.2008.02.014 18407429

[12] Tamaki T , Hirai R , Igari M , et al. Dosimetric comparison of three‐dimensional conformal radiotherapy versus volumetric‐arc radiotherapy in cervical cancer treatment: applying the central‐shielding principle to modern technology. J Radiat Res. 2018;59(5):639‐648. 10.1093/jrr/rry054 30053184 PMC6151642

